# Nurse understaffing and short work experience as predictors of healthcare-associated infections: Laura Peutere

**DOI:** 10.1093/eurpub/ckac130.150

**Published:** 2022-10-25

**Authors:** L Peutere, K Terho, J Pentti, A Ropponen, M Kivimäki, M Härmä, O Krutova, J Ervasti, A Koskinen, M Virtanen

**Affiliations:** 1 School of Educational Sciences and Psychology, University of Eastern Finland, Joensuu, Finland; 2 Department of Hospital Hygiene and Infection Control, Turku University Hospital, Turku, Finland; 3 Department of Public Health, University of Turku, Turku, Finland; 4 Finnish Institute of Occupational Health, Helsinki, Finland; 5 Division of Insurance Medicine, Karolinska Institutet, Stockholm, Sweden; 6 Faculty of Medicine, University of Helsinki, Helsinki, Finland; 7 Department of Epidemiology and Public Health, University College London, London, UK

## Abstract

**Background:**

Healthcare-associated infections (HAIs) are a serious risk factor for hospital patients leading to more than 90 000 deaths each year in European countries. It has been evaluated that 7% of patients in European acute care hospitals acquire an HAI, and that a large part of cases could be prevented. Good hand hygiene is central in preventing HAIs, which may be compromised under high work pressure. The aim of this study was to analyse the associations between nurse understaffing and short work experience with the risk of HAIs at patient-level. Prior evidence on this topic remains inconclusive due to a reliance on imprecise measurement of these exposures.

**Methods:**

We utilized administrative data on employees’ working hours and patient records from one hospital district in Finland from years 2013-2019. The data included in total 281,672 inpatient periods. We used mixed-effects survival analyses to predict the overall risk of HAIs, and four types of HAIs: bloodstream, Clostridium difficile, surgical-site and pneumonia. To consider the incubation time, exposure to nurse understaffing and short work experience were measured in preceding days in moving time windows when the patients were in the hospital.

**Results:**

Preliminary results showed that exposure to nurse understaffing within two days, measured as low nursing hours relative to planned hours, was associated with increased risk of HAIs (hazard ratio was 1.23, 95% CI 1.05-1.45). Additional analyses showed that this risk was especially pronounced in surgical-site infections, which were also carefully monitored in the hospital district. We did not find associations between exposure to short work experience among nurses and HAI risk.

**Conclusions:**

This study showed that nurse staffing below planned levels was associated with an increased overall risk of HAIs, particularly surgical-site infections, among patients. Adequate levels of nursing staff in hospitals may be important for preventing HAIs.

**Key messages:**

- Nurse understaffing may increase particularly the risk of surgical-site infections among patients.
- Nurse understaffing can be approximated based on nursing hours relative to planned hours.

